# Treatment of Biliousness

**Published:** 1883-03

**Authors:** 


DR. HARLEY in his admirable work on the Diseases of the Liver, just published, gives the following treatment of biliousness:

First give a single smart mercurial purgative. To a strong adult a powder consisting of

℞ Calomelanos.......................................... gr. iij.
Pulv. rhei............................................. gr. iv.
Magnesiæ............................................. gr. x ij.
Sig.—Take at bed time.

After the free action of this mercurial alkaline purgative in order to stimulate the secretory function of the liver and retain the bowels in as fluid a state by getting into it as much taurocholate and glycocholate of soda as possible, I prescribe half an ounce of the following mixture, to be taken in the intervals of the meals three times a day, in half a tumbler of water:

℞ Sacci taraxaci ...................................... ʒxv.

Sodii sulphates ...................................... ʒvj.

Sodii bi-carbonates ................................. ʒij.

Inf. calumbae ad .................................... ℥vj. M.

Telling the patient to shake the bottle well before measuring out his doses.

Under prophylactic treatment he says:

"The regulation of the patient's food, drink and exercise is therefore the first—I might almost say the chief—thing requiring to be considered; for, with a well regulated diet and régime, attacks of biliousness might be reduced to things almost unknown."

It is not only the quality but the quantity of the food that has to be regulated. For an excess of the most wholesome of foods acts in persons predisposed to biliousness as an exciting cause. Therefore it is a good plan never to allow the patient at any time to eat more than the wants of his system demand. If he is corpulent even put him on short commons. Stop his beer and reduce his wine to a couple glasses of hock or claret a day. If he be thin, or only moderately stout, only stop all salted food, ham, bacon, hung beef, haddock, etc. Order him to take nothing but fresh food—not too fatty, and rather underdone; to avoid shell fish and pastry; to make his chief meal in the middle of the day; to take as much walking exercise as he possibly can without fatiguing himself, and to go to bed early." * *

"In some persons with a more than usual tendency to biliousness, traceable to biliary secretion, besides chronic hepatic capillary congestion there sometimes exists also defective nerve action. The secretory nerve twigs appear as if they had literally as well as figuratively gone to sleep, and I believe it is by waking them up, as it were, with small doses of nux vomica, that homœopathic practitioners have obtained the credit of being able to cure bad bilious attacks. Certainly, for some years past, I have taken a leaf out of their book, and treated many cases of torpid liver with strychnia with marked benefit. The rationale of the action of the strychnia in these cases appears to me to be precisely the same that it is in mild cases of paralysis, where it evidently calls nerve action into play, as is visibly seen by the paralyzed muscles twitching under its influence. When given in torpid liver from defective nerve influence, the stimulating effect of the strychnia upon the nerve is, I think, rendered equally patent to our minds, though not to our eyes, by the increase of the biliary secretion.

Moreover, as a sluggish state of the bowels is a usual concomitant of a sluggish biliary secretion, I usually combine the strychnia with belladonna, which has a specific action on the intestines, and produces easy motions without purging the patient.

In order to gain a still further advantage, I give, as a rule, the strychnia and belladonna in combination with taraxacum, generally in the form of a dinner pill, to be taken at the commencement of the meal; and, as I should like others to try my plan, its formula is as follows:

| | | |
|---|---|---|
| ℞ | Strychniæ acetatis ............................ | gr. j. |
| | Ext. belladonnæ.................................. | gr. vj. |
| | Aloes socotrinæ.................................. | gr. xiv. |
| | Ext. taraxaci ..................................... | ʒij.—M. |
| | Divide in pil....................................... | xxxvj. |

In another place he says that many of the liver cases, especially those met with among women, are due in a great measure to the pernicious, and also, now-a-days, but too common practice of flying to sleeping draughts and soothing mixtures on every trifling occasion; for nothing in the world so effectively and promptly impedes the performance of the hepatic function as narcotics be they opiate or chlorals. A couple of grains of opium will bring on an attack of biliousness more speedily in most persons than anything else; while its prolonged use will lead to active as well as passive congestions of the liver.

